# Synergistic anticancer and antibacterial effects of novel regimens of phytopolyphenols and repurposing drugs on cultured cells

**DOI:** 10.32604/or.2025.063717

**Published:** 2025-06-26

**Authors:** YA-LING YEH, YING-JAN WANG, SHOEI-YN LIN-SHIAU

**Affiliations:** 1 Department of Environmental and Occupational Health, College of Medicine, National Cheng Kung University, Tainan, 70428, Taiwan; 2 School of Dentistry, College of Oral Medicine, Chung Shan Medical University, Taichung, 40201, Taiwan; 3 Department of Pharmacology, College of Medicine, National Taiwan University, Taipei, 10051, Taiwan

**Keywords:** Novel regimens, Phytopolyphenols, Repurposing drugs, Anticancer, ATPase inhibition, Antibacterial

## Abstract

**Background:**

The increasing incidence of cancers and infectious diseases worldwide presents a significant public health challenge that requires immediate intervention. Our strategy to tackle this issue involves the development of pharmaceutical formulations that combine phytopolyphenols (P), targeted drugs (T), and metal ions (M), collectively referred to as PTM regimens. The diverse pharmacological properties of PTM regimens are hypothesized to effectively reduce the risk factors associated with both cancers and infectious diseases.

**Methods:**

The effects of the pharmaceutical agents on the proliferation of cultured cancer cells and pathogens were assessed after 72 h and 48 h, respectively, using the MTT (3-[4,5-dimethylthiazol-2-yl]-2,5 diphenyl tetrazolium bromide) assay and optical density at 600 nm (OD600). The synergistic effects of drug combinations were evaluated by combination index (CI), where CI < 1 indicates synergism, CI = 1 indicates addition, and CI > 1 indicates antagonism. Efficacy index (EI) was also calculated. Assays of efflux pump ATPase activities were conducted using a colorimetric method.

**Results:**

This study evaluated the anticancer and antibacterial efficacy of PTM regimens that included phytopolyphenols (specifically curcumin (C) and green tea polyphenols (G)), repurposed drugs (memantine (Mem), thioridazine (TRZ), cisplatin (Cis), and 5-fluorouracil (5FU)), and ZnSO_4_ (Zn) across three cultured cancer cell lines and four cultured pathogens. The most effective regimens, GC•Mem•Zn and GC•TRZ•Zn, significantly enhanced the anticancer efficacy (EI) of cisplatin across the three cancer lines (OECM-1, A549 and DLD-1) by 7, 11 and 21; 7, 9, and 17 fold, respectively, while the enhancements for 5-fluorouracil were 5, 6 and 12; 5, 5 and 9 fold, respectively. Furthermore, these PTM regimens demonstrated substantial synergistic inhibition of Na^+^-K^+^-Mg^2+^-ATPase and Mg^2+^-ATPase in the cultured cancer cells, as well as a reduction in biofilm formation by the four cultured pathogens, suggesting their potential to address the challenges of multidrug resistance in cancers and infectious diseases.

**Conclusion:**

Given that all drugs incorporated in the PTM regimens have been clinically validated for safety and efficacy, particularly regarding their synergistic selective anticancer efficacy, inhibition of efflux pump ATPase, and antibiofilm formation of pathogens, these regimens may offer a promising therapeutic strategy to alleviate the severe side effects and drug resistance typically associated with chemotherapeutic agents. Further preclinical and clinical investigations are warranted.

## Introduction

The global increase in multidrug-resistant cancers and infectious diseases presents substantial challenges that require immediate intervention [1]. Various risk factors associated with carcinogenesis include oxidative stress, inflammation, immunodeficiency, mitochondrial dysfunction, and gut dysbiosis [2]. In response to these pressing issues, we have developed pharmaceutical compositions that integrate phytopolyphenols (P), targeted drugs (T), and metal ions (M), collectively termed PTM regimens. These regimens are designed to prevent and manage both cancers and infectious diseases.

Key findings related to PTM regimens highlight their diverse pharmacological effects, which encompass: (1) antioxidant properties; (2) anti-inflammatory effects; (3) anticancer activity; (4) antibacterial properties; (5) neuroprotection; and (6) mitochondrial and immunomodulatory effects [3–5]. These regimens have been recognized as innovative therapeutic strategies, resulting in the issuance of five patents for their applications in preventing and managing cancers and various chronic diseases [6], including neurodegenerative diseases, dementia [7], and chronic pain [8]. This study aims to explore the synergistic anticancer and antibacterial effects of selected PTM regimens.

The pharmacological agents utilized in this study include phytopolyphenols (P), such as curcumin (C) and green tea polyphenols (G); targeted drugs (T), including memantine (Mem), thioridazine (TRZ), cisplatin (Cis), and 5-fluorouracil (5FU), as well as zinc sulfate (Zn). These repurposed drugs have demonstrated clinical efficacy and safety. Memantine is prescribed for dementia [9,10], while thioridazine is indicated for neurodegenerative diseases [11,12]; both cisplatin [13,14] and 5FU [15,16] are among the most frequently employed chemotherapeutic agents. However, these treatments can result in severe adverse effects, including nephrotoxicity, neurotoxicity, gastrointestinal disturbances, and oral mucositis, which affects 60–80% of patients, as well as dysbiosis, potentially leading to the discontinuation of chemotherapy [16,17]. Furthermore, the challenges posed by inherent or acquired multidrug resistance are considerable.

The issue of multidrug resistance (MDR) in cancers and infectious diseases represents a significant problem that necessitates resolution. Recent studies have indicated that a synergistic alternative strategy involving drug repurposing may be effective in the treatment of cancers and infectious diseases [18–20]. For instance, memantine combined with high doses of vitamin D [21] or donepezil [22] has demonstrated synergistic effects in the treatment of Alzheimer’s disease. Similarly, thioridazine in conjunction with antibiotics [11] or moxifloxacin [23] or tetracycline [24] has been effective in treating drug-resistant infections such as pulmonary tuberculosis or exhibiting synergistic antibacterial effects, respectively. This suggests a promising avenue for exploring the therapeutic potential of combinatorial drug repurposing. In alignment with these contemporary approaches, our study demonstrates the synergistic effects and enhanced selectivity of novel PTM regimens against cancers and infectious diseases, as evidenced by experiments conducted on cultured oral (OECM-1), lung (A549), and colon (DLD-1) cancer cells, as well as on four cultured pathogenic biofilms. The potential of PTM regimens to address the rising prevalence of cancers and infectious diseases warrants further clinical investigation.

## Materials and Methods

### Drugs studied

Curcumin (C) (#8.20354) was purchased from Merck Co. (Darmstadt, Germany). Green tea polyphenols, purified from *Camellia sinensis* and containing 98% polyphenols (75% catechins by HPLC and 50% EGCG by HPLC), similar to polyphenol E, were obtained from Hunan Huacheng Biotech, Inc. (China). ZnSO_4_ (#204986), Cisplatin (#232120), memantine (#5.04905), and thioridazine (#T9025) were sourced from Sigma Chemical Company (St. Louis, MO, USA). 5-Fluorouracil was acquired from the Department of Pharmacy at Chung-Shan Medical University Hospital (Taichung, Taiwan).

### Cell culture

The cell culture medium utilized was RPMI-1640 (#R8758), along with necessary supplements, which were obtained from Sigma (St. Louis, MO, USA). All culture media were supplemented with heat-inactivated 10% fetal bovine serum (FBS) sourced from either JRH Biosciences or Hyclone, Thermo Scientific and penicillin (62.5 μg/mL)/streptomycin (100 μg/mL). The following cell lines were utilized: SG (Smulow-Glinckman human normal gingival epithelial cells), OECM-1 (oral epidermoid carcinoma meng-1 cell), A549 (ATCC No.CCL-185 human *RAS* mutated non-small cell lung cancer cells), and DLD-1 (ATCC No. CCL-221 human *p53* mutated colon cancer cells), all of which were obtained from American Type Culture Collection (ATCC, Manassas, VA, USA). The mycoplasma test of all these cell cultures by ATCC revealed negative. All of the cell lines were cultured in accordance with the supplier’s instructions. Culturing was conducted in a CO_2_ incubator maintained at a concentration of 5% and a temperature of 37 ± 0.5°C.

### The culture of pathogens

The anaerobic pathogen *Porphyromonas gingivalis* (P.g., ATCC 33277) was cultured in Luria-Bertani (LB) broth (#244620, Becton Dickinson, Sparks, MD) supplemented with hemin(#H9039) (5 μg/mL) and menadione (#NC1528913) (1 μg/mL) (Sigma-Aldrich, St. Louis, Missouri, USA). *Streptococcus mutans* (S.m., UA159, ATCC 700610) was cultured in Brain Heart Infusion (BHI) broth (#256120, Becton Dickinson, Sparks, MD). Both P.g. and UA159 exhibit growth at a temperature of 37 ± 0.5°C within an anaerobic chamber containing 10% H_2_, 5% CO_2_, and 85% N_2_ (Forma Scientific Inc., Marietta, OH, USA). *Pseudomonas aeruginosa* (P.a., ATCC 39327) was cultured in LB, and *Staphylococcus aureus* (S.a., ATCC 33591) was cultured in BHI. Both P.a. and S.a. exhibit growth at a temperature of 37 ± 0.5°C under aerobic conditions.

### Anticancer and antibacterial potencies (IC_50_)

Cells were cultured in a 96-well microplate, with each well containing 6 × 10^3^ cells in 100 μL of medium. Following a 24-hour incubation period, 10 μL of the drug solution was introduced into each well, and the cells were subsequently incubated for an additional 72 h. Cell proliferation was evaluated using the MTT (3-[4,5-dimethylthiazol-2-yl]-2,5-diphenyl tetrazolium bromide) assay, with absorbance measured at OD550 using a BioTek Epoch microplate spectrophotometer (Agilent Technologies, Inc., Santa Clara, CA, USA) [25].

Bacterial cultures were diluted to achieve an optical density (OD600) of 0.04–0.06, corresponding to approximately 2 × 10^8^ CFU/mL, and were then subcultured in a 96-well microplate. The bacterial strains were diluted in specific culture broths: *Porphyromonas gingivalis* (P.g.) in LB medium supplemented with hemin and menadione, *Streptococcus mutans* (UA159) in brain heart infusion (BHI) supplemented with 0.2% sucrose, *Pseudomonas aeruginosa* (P.a.) in LB, and *Staphylococcus aureus* (S.a.) in BHI supplemented with 10% human plasma, with 100 μL per well. Various concentrations of the drugs were added at 10 μL per well, and the cultures were incubated at 37 ± 0.5°C for 48 h to facilitate biofilm formation. The antibacterial efficacy of the drugs was assessed after the 48-h incubation period by measuring OD600. The potency (IC_50_, defined as the concentration required for 50% inhibition of biofilm formation) was determined using the concentration-inhibition curve as previously described [25].

### Assay of ATPase activity

ATPase activities were assessed using a colorimetric method as previously described [26]. Briefly, after treatment with drugs for 72 h, the cells were incubated with the substrate ATP for 15 min at 37 ± 0.5°C. The released inorganic phosphate (Pi) was quantified by measuring the optical density at 630 nm (OD630) after a 10-min reaction with malachite green-ammonium molybdate color reagents (1 volume of 4.2% ammonium molybdate tetrahydrate (#M1019, Merck, Darmstadt, Germany) in 4N HCl, added 3 volumes of 0.045% malachite green carbinol hydrochloride (#213020, Merck, Darmstadt, Germany)). A standard curve for Pi was established simultaneously.

### Synergistic effect analysis

The synergistic effect (CI < 1) was calculated using the combination index (CI) equation [27]:

$$\begin{array}{rl} CI=\; & \frac{{\left(IC_{50}\right)}_{1}\;in\;combination}{{\left(IC_{50}\right)}_{1}\;alone}+\frac{{\left(IC_{50}\right)}_{2}\;in\;combination}{{\left(IC_{50}\right)}_{2}\;alone}\\ & +\frac{{\left(IC_{50}\right)}_{3}\;in\;combination}{{\left(IC_{50}\right)}_{3}\;alone} \end{array}$$

$$\begin{array}{r} CI\;<\;1,synergism;CI=1,addition;CI\;>\;1,antagonism \end{array}$$

The efficacy of anticancer or antibacterial effects was determined using the efficacy index (EI):

$$EI=\frac{{\left(IC_{50}\right)}_{alone}}{{\left(IC_{50}\right)}_{combination}}$$

EI > 1,synergism;EI=1,addition;EI < 1,antagonism

### Statistical analysis

All experiments were conducted in triplicate and repeated three times (n = 3). The results for each experiment are expressed as mean ± standard error of the mean (SEM). A one-way analysis of variance (ANOVA) followed by a post-hoc *t*-test (Microsoft Excel, 2019) was utilized to evaluate the differences among the groups. The statistical significance of the differences was determined, with a significance threshold established at *p* < 0.05.

## Results

### Anticancer effects

**Figure 1:**
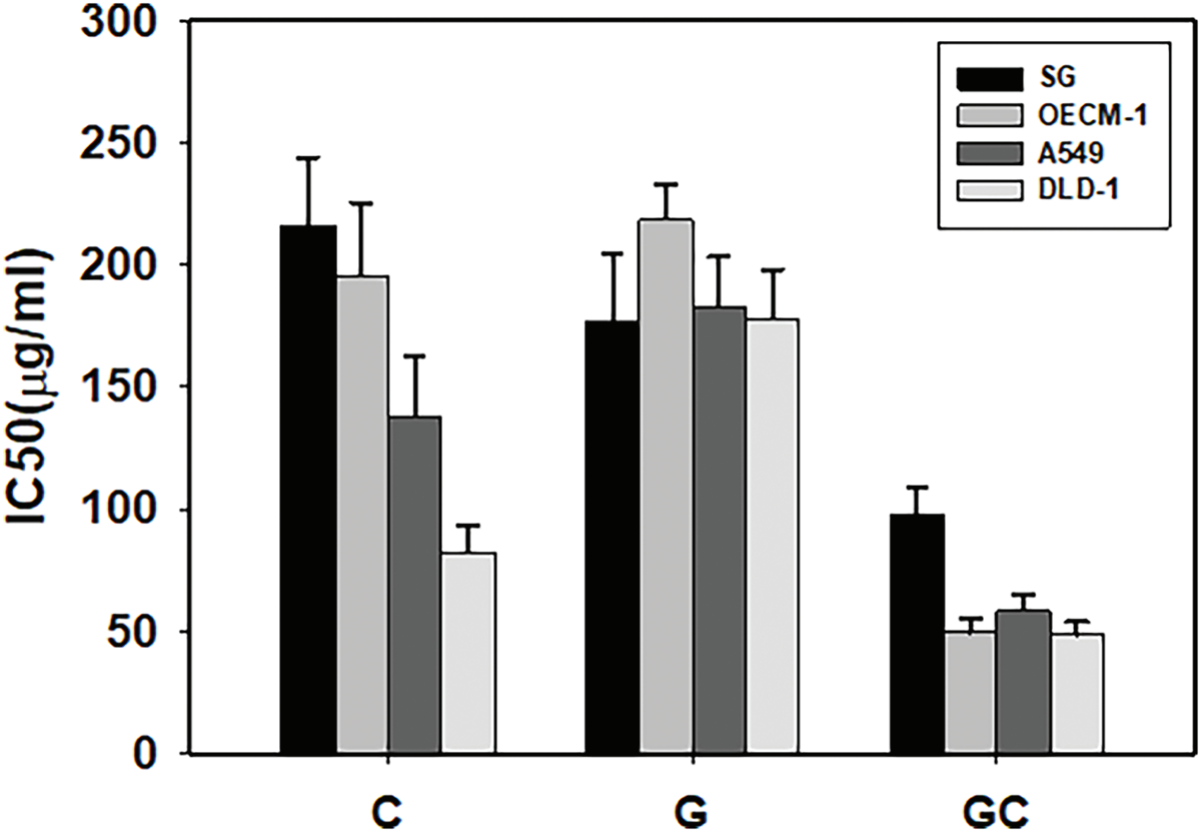
Anti-proliferative potencies (IC_50_) of drugs administered individually on cultured cells. C, curcumin; G, green tea polyphenols; GC, green tea polyphenols and curcumin combination. SG, gingival epithelial cell; OECM-1, oral cancer cell; A549, lung cancer cell; DLD-1, colon cancer cell.

The anticancer efficacy, as indicated by the IC_50_ values, of the drugs administered individually (A) and in combination with PTM regimens (B) on cultured cancer cell lines (OECM-1, A549, and DLD-1) was compared to their effects on normal gingival epithelial cells (SG), as presented in Table 1 and Fig. 1. Both C and GC demonstrated selective anticancer properties, as evidenced by their IC_50_ values being lower in cancer cells than in SG cells, whereas G and ZnSO_4_ did not exhibit this selectivity.

**Table 1:** Anti-cancer potency (IC_50_^†^) of drugs administered individually (A) and PTM regimens (B) on cultured cells

(A) Drug alone				
	^†^IC_50_ (μg/mL; Zn^2+^, μM)			
Drugs	SG	OECM-1	A549	DLD-1
C, Curcumin	215.9 ± 28.1	195.2 ± 30.3	137.9 ± 24.9	82.3 ± 10.9
G, Green tea polyphenol	176.4 ± 20.2	218.4 ± 14.6	182.6 ± 20.9	178.0 ± 19.9
Zn, ZnSO_4_	267.8 ± 7.9	405.1 ± 15.9	561.8 ± 12.8	295.0 ± 19.8
Cis, Ciaplatin	6.5 ± 0.4	5.8 ± 0.5	10.1 ± 0.8	8.4 ± 1.0
5FU, 5-Fluorouracil	3.1 ± 0.3	4.3 ± 0.3	3.5 ± 0.3	3.6 ± 0.3
Mem, Memantine	55.2 ± 2.8	115.6 ± 7.8	108.4 ± 6.6	61.7 ± 3.8
TRZ, Thioridazine	8.4 ± 0.8	11.7 ± 1.3	10.6 ± 1.3	9.2 ± 1
^†^IC_50_, the concentration (μg/mL) required for 50% inhibition of the growth of cultured cells. SG, Gingival epithelial cell; OECM-1, Oral cancer cell; A549, Lung cancer cell; DLD-1, Colon cancer cell.				

(B) PTM regimens						
Drugs	SG			OECM-1		
	IC_50_ (μg/mL; Zn^2+^, μM)	CI^†^	EI^‡^	IC_50_ (μg/mL; Zn^2+^, μM)	CI	EI
G•C	98.1 ± 10.9•98.1 ± 10.9	1.0		49.5 ± 5.9•49.5 ± 5.9	0.5	
G•C•Zn	54.6 ± 4.1•54.6 ± 4.1•18.2 ± 1.4	0.6		49.0 ± 3.9•49.0 ± 3.9•16.3 ± 1.3	1.0	
C•Mem•Zn	23.9 ± 1.4•23.9 ± 1.4•8.0 ± 0.5	0.6	2*	45.8 ± 4.3•45.8 ± 4.3•15.3 ± 1.4	0.7	3*
C•TRZ•Zn	23.1 ± 2.6•2.3 ± 0.3•7.7 ± 0.9	0.5	4*	35.3 ± 2.6•3.5 ± 0.3•11.8 ± 0.9	0.6	3*
G•C•Mem•Zn	31.4 ± 1.5•31.4 ± 1.5•31.4 ± 1.5•10.5 ± 0.5	0.9	2*	26.6 ± 1.5•26.6 ± 1.5•26.6 ± 1.5•8.9 ± 0.5	0.8	4*
G•C•TRZ•Zn	29.1 ± 1.3•29.1 ± 1.3•2.9 ± 0.1•9.7 ± 0.4	0.7	3*	30.9 ± 2.4•30.9 ± 2.4•3.1 ± 0.2•10.3 ± 0.8	0.9	4*

Drugs	A549			DLD-1		
	IC_50_ (μg/mL; Zn^2+^, μM)	CI	EI	IC_50_ (μg/mL; Zn^2+^, μM)	CI	EI
G•C	58.3 ± 6.8•58.3 ± 6.8	0.7		48.8 ± 5.2•48.8 ± 5.2	0.9	
G•C•Zn	97.6 ± 8.4•97.6 ± 8.4•32.5 ± 2.8	1.7		59.7 ± 4.5•59.7 ± 4.5•19.9 ± 1.5	1.3	
C•Mem•Zn	66.5 ± 4.9•66.5 ± 4.9•22.1 ± 1.6	1.1	2*	18.9 ± 1.3•18.9 ± 1.3•6.3 ± 0.4	0.6	3*
C•TRZ•Zn	42.3 ± 4.1•4.2 ± 0.4•14.1 ± 1.4	0.8	3*	17.5 ± 1.5•1.7 ± 0.1•5.8 ± 0.5	0.5	5*
G•C•Mem•Zn	35.0 ± 2.1•35.0 ± 2.1•35.0 ± 2.1•11.7 ± 0.7	0.9	3*	35.7 ± 2.6•35.7 ± 2.6•35.7 ± 2.6•11.9 ± 0.9	1.4	2*
G•C•TRZ•Zn	30.8 ± 1.7•30.8 ± 1.7•3.1 ± 0.2•10.3 ± 0.6	0.8	3*	16.2 ± 1.3•16.2 ± 1.3•1.6 ± 0.1•5.4 ± 0.4	0.5	6*
^†^CI (combination index) and ^‡^EI (efficacy index) were calculated using the equations described in the Methods section. EI of Mem or TRZ in each regimen is indicated. *The increased EI values are statistically significants *p* < 0.05.						

**Figure 2:**
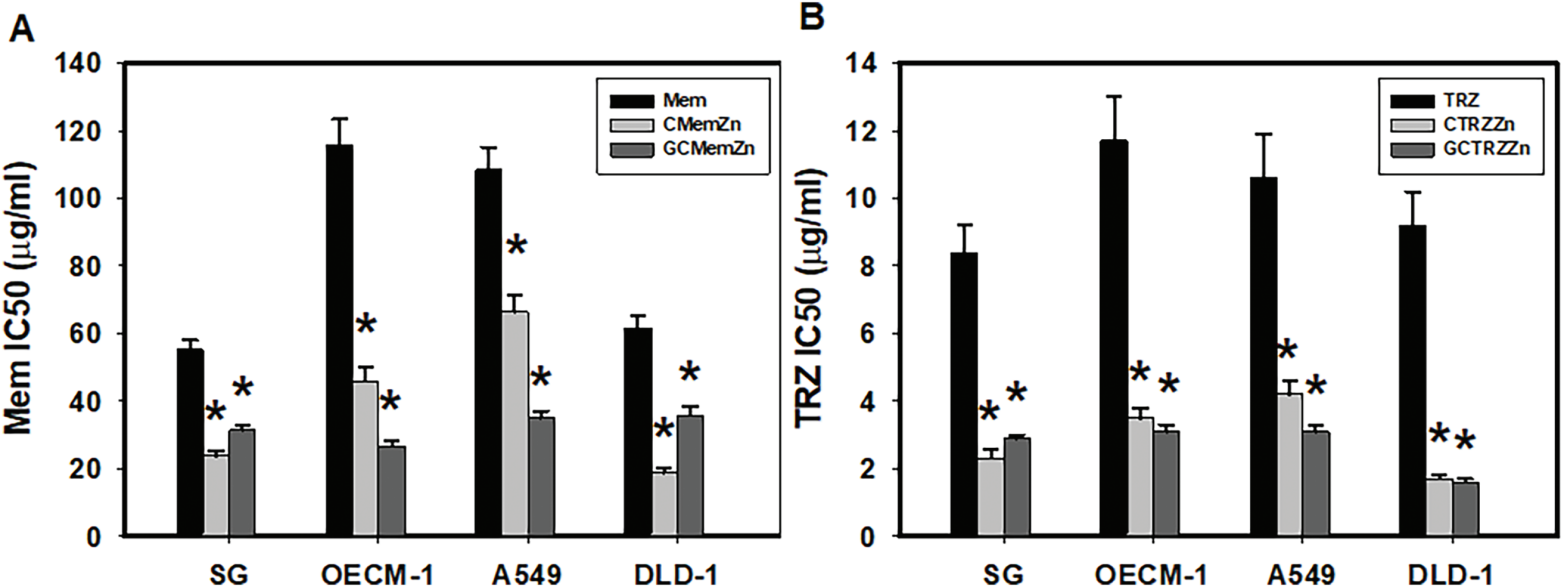
Anti-proliferative potencies (IC_50_) of Mem (memantine), CMemZn and GCMemZn (A) and TRZ (thioridazine) (B), CTRZZn and GCTRZZn on cultured cells. *p < 0.05.

Table 1B and Fig. 2 illustrate the anticancer potency (IC_50_), synergistic interactions (CI < 1), and enhanced efficacy (EI > 1) of the PTM regimens that included memantine (Mem) and thioridazine (TRZ). The majority of these regimens exhibited synergistic anticancer efficacy (CI < 1) alongside increased efficacy (EI > 1). Notably, the combination of GC•Mem(TRZ)•Zn displayed superior selective and synergistic anticancer effects compared to the administration of Mem or TRZ alone.

**Figure 3:**
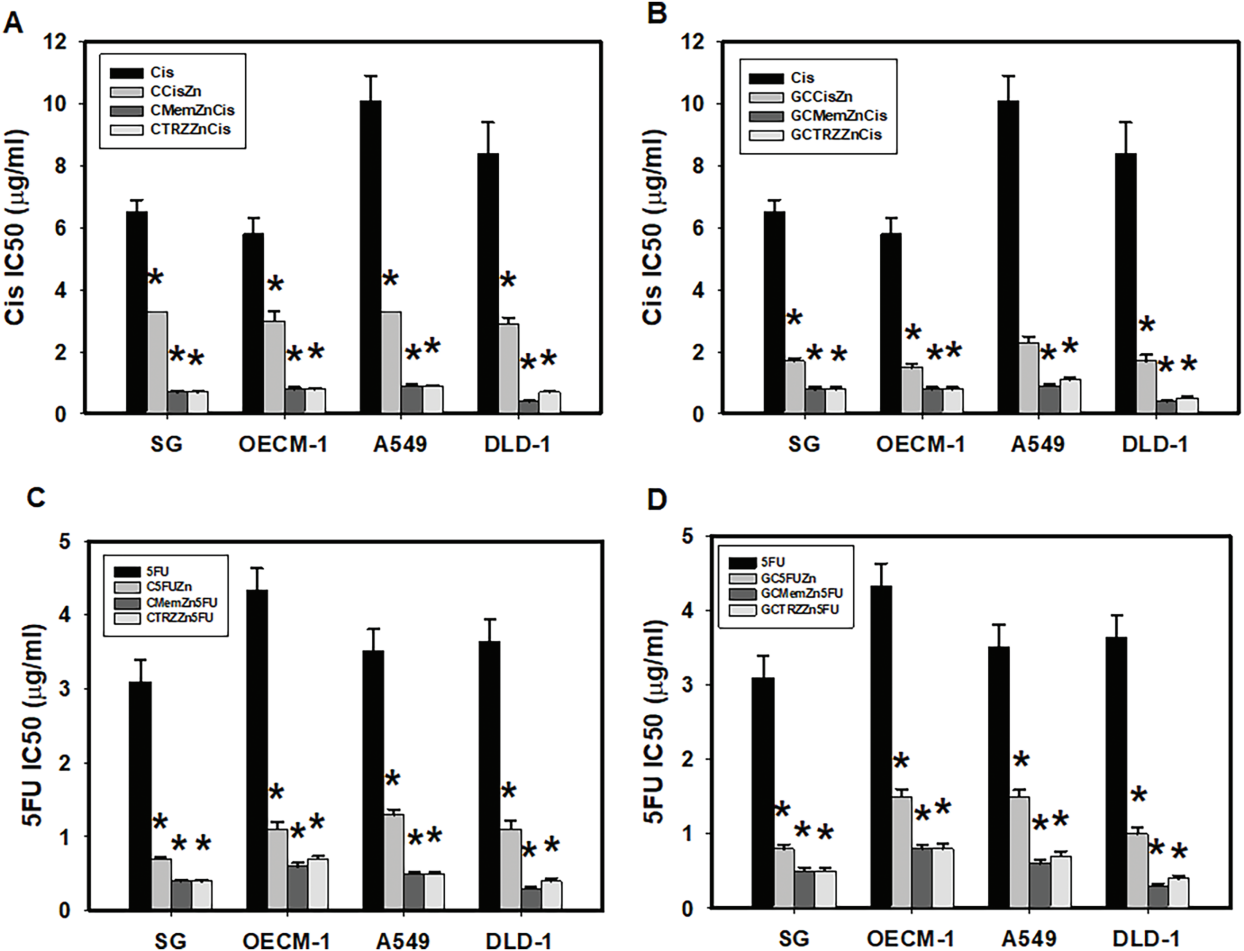
PTM regimens synergistically enhanced selective anticancer effects of cisplatin (Cis, A, B) and 5-fluorouracil (5FU, C, D) on cultured cells. *p < 0.05.

Moreover, these PTM regimens significantly enhanced the selective anticancer efficacy of Cisplatin (Cis) and 5-Fluorouracil (5FU) in a synergistic manner (CI < 1), as detailed in Table 2 and Fig. 3. The most effective regimens, GC•Mem•Zn and GC•TRZ•Zn, significantly enhanced the anti-cancer efficacy (EI) of Cis across the three cancer lines (OECM-1, A549 and DLD-1) by 7, 11 and 21 (Table 2A and Fig. 3B); 7, 9, and 17 fold, respectively, while the enhancements for 5FU were 5, 6 and 12; 5, 5 and 9 fold, respectively (Table 2B and Fig. 3D). Similarly, the C•Mem•Zn combination yielded increases of 7, 11 and 12 fold efficacy of Cis (Table 2A and Fig. 3A) and 7, 7, and 12 fold in the efficacy of 5FU against the same cancer cell lines (Table 2B and Fig. 3C).

**Table 2:** PTM regimens synergistically enhance (CI < 1) selective anticancer efficacy (EI > 1) of cisplatin (A) and 5-fluorouracil (B) on cultured cancer cells

(A) Cisplatin						
Drugs	SG			OECM-1		
	IC_50_ (μg/mL; Zn^2+^, μM)	CI	EI^†^	IC_50_ (μg/mL; Zn^2+^, μM)	CI	EI^†^
C•Cis•Zn	>100•3.3•33.3	1.1	2	90.6 ± 8.5•3.0 ± 0.3•30.2 ± 2.8	1.1	2*
C•Mem•Zn•Cis	20.3 ± 1.1•20.3 ± 1.1•6.8 ± 0.4•0.7 ± 0.04	0.6	9*	23.1 ± 2.0•23.1 ± 2.0•7.7 ± 0.7•0.8 ± 0.07	0.5	7*
C•TRZ•Zn•Cis	21.1 ± 1.0•2.1 ± 0.1•7.0 ± 0.3•0.7 ± 0.03	0.5	9*	22.8 ± 1.3•2.3 ± 0.1•7.6 ± 0.4•0.8 ± 0.04	0.5	7*
G•C•Cis•Zn	49.8 ± 3.4•49.8 ± 3.4•1.7 ± 0.1•16.6 ± 1.1	0.8	4*	45.0 ± 3.7•45.0 ± 3.7•1.5 ± 0.1•15.0 ± 1.2	1.2	4*
G•C•Mem•Zn•Cis	23.2 ± 1.7•23.2 ± 1.7•23.2 ± 1.7•7.7 ± 0.6•0.8 ± 0.06	0.8	8*	23.1 ± 1.6•23.1 ± 1.6•23.1 ± 1.6•7.7 ± 0.5•0.8 ± 0.05	0.8	7*
G•C•TRZ•Zn•Cis	23.9 ± 2.0•23.9 ± 2.0•2.4 ± 0.2•8.0 ± 0.66•0.8 ± 0.07	0.7	8*	23.1 ± 1.9•23.1 ± 1.9•2.3 ± 0.19•7.7 ± 0.64•0.8 ± 0.06	0.8	7*

Drugs	A549			DLD-1		
	IC_50_ (μg/mL; Zn^2+^, μM)	CI	EI^†^	IC_50_ (μg/mL; Zn^2+^, μM)	CI	EI^†^
C•Cis•Zn	>100•3.3•33.3	1.1	3*	88.8 ± 6.1•2.9 ± 0.2•29.6 ± 2.0	1.5	3*
C•Mem•Zn•Cis	26.1 ± 1.5•26.1 ± 1.5•8.7 ± 0.5•0.9 ± 0.05	0.5	11*	11.7 ± 0.6•11.7 ± 0.6•3.9 ± 0.2•0.4 ± 0.02	0.4	21*
C•TRZ•Zn•Cis	26.1 ± 0.9•2.6 ± 0.09•8.7 ± 0.3•0.9 ± 0.03	0.5	11*	19.9 ± 1.1•2.0 ± 0.1•6.6 ± 0.4•0.7 ± 0.04	0.6	12*
G•C•Cis•Zn	69.3 ± 6.4•69.3 ± 6.4•2.3 ± 0.2•23.1 ± 2.1	1.5	4	50.1 ± 5.8•50.1 ± 5.8•1.7 ± 0.2•16.7 ± 1.9	1.3	5*
G•C•Mem•Zn•Cis	26.3 ± 1.9•26.3 ± 1.9•26.3 ± 1.9•8.8 ± 0.6•0.9 ± 0.06	0.8	11*	11.5 ± 0.8•11.5 ± 0.8•11.5 ± 0.8•3.8 ± 0.6•0.4 ± 0.03	0.5	21*
G•C•TRZ•Zn•Cis	31.6 ± 2.0•31.6 ± 2.0•3.2 ± 0.2•10.5 ± 0.7•1.1 ± 0.07	1.0	9*	14.9 ± 1.5•14.9 ± 1.5•1.5 ± 0.15•5.0 ± 0.5•0.5 ± 0.05	0.5	17*
^†^EI of Cis in each regimen is indicated. *The increased EI values are statistically significants *p* < 0.05.						

(B) 5-Fluorouracil						
Drugs	SG			OECM-1		
	IC_50_ (μg/mL; Zn^2+^, μM)	CI	EI^†^	IC_50_ (μg/mL; Zn^2+^, μM)	CI	EI^†^
C•5FU•Zn	22.1 ± 0.98•0.7 ± 0.03•7.4 ± 0.3	0.4	4*	33.8 ± 3.5•1.1 ± 0.1•11.3 ± 1.2	0.5	4*
C•Mem•Zn•5FU	11.0 ± 0.6•11.0 ± 0.6•3.7 ± 0.2•0.4 ± 0.02	0.4	8*	18.9 ± 1.4•18.9 ± 1.4•6.3 ± 0.5•0.6 ± 0.05	0.4	7*
C•TRZ•Zn•5FU	12.8 ± 0.6•1.3 ± 0.06•4.3 ± 0.2•0.4 ± 0.02	0.4	8*	19.9 ± 1.3•2.0 ± 0.13•6.6 ± 0.4•0.7 ± 0.04	0.5	6*
G•C•5FU•Zn	23.7 ± 1.7•23.7 ± 1.7•0.8 ± 0.06•7.9 ± 0.6	0.5	4*	45.0 ± 2.9•45.0 ± 2.9•1.5 ± 0.1•15.0 ± 1.0	1.3	3*
G•C•Mem•Zn•5FU	16.1 ± 1.6•16.1 ± 1.6•16.1 ± 1.6•5.4 ± 0.5•0.5 ± 0.05	0.6	6*	24.8 ± 1.6•24.8 ± 1.6•24.8 ± 1.6•8.3 ± 0.5•0.8 ± 0.05	0.9	5*
G•C•TRZ•Zn•5FU	15.6 ± 1.4•15.6 ± 1.4•1.6 ± 0.14•5.2 ± 0.45•0.5 ± 0.05	0.5	6*	24.7 ± 2.0•24.7 ± 2.0•2.5 ± 0.2•8.2 ± 0.65•0.8 ± 0.07	0.9	5*

Drugs	A549			DLD-1		
	IC_50_ (μg/mL; Zn^2+^, μM)	CI	EI^†^	IC_50_ (μg/mL; Zn^2+^, μM)	CI	EI^†^
C•5FU•Zn	37.9 ± 2.1•1.3 ± 0.07•12.6 ± 0.7	0.7	3*	32.1 ± 3.6•1.1 ± 0.12•10.7 ± 1.2	0.7	3*
C•Mem•Zn•5FU	15.9 ± 1.0•15.9 ± 1.0•5.3 ± 0.3•0.5 ± 0.03	0.4	7*	9.1 ± 0.5•9.1 ± .5•3.0 ± 0.2•0.3 ± 0.02	0.4	12*
C•TRZ•Zn•5FU	16.1 ± 1.0•1.6 ± 0.1•5.4 ± 0.3•0.5 ± 0.03	0.4	7*	13.5 ± 1.1•1.3 ± 0.11•4.5 ± 0.4•0.4 ± 0.04	0.4	9*
G•C•5FU•Zn	46.2 ± 2.8•46.2 ± 2.8•1.5 ± 0.09•15.4 ± 0.9	1.2	2*	31.2 ± 2.7•31.2 ± 2.7•1.0 ± 0.09•10.4 ± 0.9	0.9	4*
G•C•Mem•Zn•5FU	17.6 ± 1.5•17.6 ± 1.5•17.6 ± 1.5•5.9 ± 0.5•0.6 ± 0.05	0.6	6*	9.9 ± 0.9•9.9 ± 0.9•9.9 ± 0.9•3.3 ± 0.3•0.3 ± 0.03	0.5	12*
G•C•TRZ•Zn•5FU	22.2 ± 2.0•22.2 ± 2.0•2.2 ± 0.20•7.4 ± 0.67•0.7 ± 0.07	0.8	5*	12.5 ± 1.2•12.5 ± 1.2•1.3 ± 0.12•4.2 ± 0.4•0.4 ± 0.04	0.5	9*
^†^EI of 5FU in each regimen is indicated. *The increased EI values are statistically significants *p* < 0.05.						

### ATPase inhibitors on cancer cell lines

Table 3 presents the inhibitory effects of various pharmacological agents on Na^+^-K^+^-Mg^2+^-ATPase (A) and Mg^2+^-ATPase (B) activities in cultured cancer cells. Analysis of IC_50_ values indicates that all tested agents, including G, Zn, 5FU, Mem, and TRZ, exhibited selective inhibition of Na^+^-K^+^-Mg^2+^-ATPase activity in DLD-1 cells. In contrast, only G and TRZ demonstrated selective inhibition in OECM-1 cells, while G and 5FU were effective in A549 cells. Conversely, all agents, with the exception of 5FU and Mem, selectively inhibited Mg^2+^-ATPase activity in OECM-1 cells, but not in DLD-1 cells. Additionally, C and TRZ selectively inhibited Mg^2+^-ATPase activity in A549 cells.

**Table 3:** Potencies (IC_50_) of ATPase inhibition by drugs administered individually on cultured cells. (A) Na^+^-K^+^-Mg^2+^-ATPase inhibition; (B) Mg^2+^-ATPase inhibition

(A)				
	IC_50_ (μg/mL; Zn^2+^, μM)			
Drugs	SG	OECM-1	A549	DLD-1
C, Curcumin	33.8 ± 2.3	58.7 ± 7.4	323.6 ± 15.6	200.6 ± 23.5
G, Green tea polyphenol	80.8 ± 2.4	50.8 ± 2.5	66.1 ± 4.5	58.5 ± 3.0
Zn, ZnSO_4_	321.5 ± 36.5	396.9 ± 30.2	426.1 ± 43.0	299.1 ± 36.6
G•C	62.2 ± 7.0•62.2 ± 7.0	53.3 ± 3.2•53.3 ± 3.2	75.0 ± 8.4•75.0 ± 8.4	69.2 ± 6.8•69.2 ± 6.8
G•C•Zn	41.3 ± 1.9•41.3 ± 1.9•13.7 ± 0.6	22.7 ± 2.8•22.7 ± 2.8•7.5 ± 0.9	63.0 ± 3.4•63.0 ± 3.4•21.0 ± 1.1	34.9 ± 2.1•34.9 ± 2.1•11.6 ± 0.7
Cis, Ciaplatin	2.6 ± 0.9	3.3 ± 0.6	4.6 ± 1.3	3.9 ± 0.5
5FU, 5-Fluorouracil	5.2 ± 0.3	10.8 ± 1.1	4.0 ± 0.2	4.4 ± 0.2
Mem, Memantine	61.1 ± 4.0	105.8 ± 9.4	81.4 ± 7.6	55.6 ± 5.2
TRZ, Thioridazine	7 ± 0.7	7.5 ± 1.0	10.2 ± 1.4	7.7 ± 0.3

(B)				
	IC_50_ (μg/mL; Zn^2+^, μM)			
Drugs	SG	OECM-1	A549	DLD-1
C, Curcumin	67.1 ± 5.5	62.0 ± 5.8	170.5 ± 16.4	323.8 ± 15.0
G, Green tea polyphenol	88.9 ± 5.5	80.9 ± 8.5	105.1 ± 12.4	133.9 ± 9.0
Zn, ZnSO_4_	248.6 ± 19.2	244.4 ± 23.1	310.1 ± 19.9	293.6 ± 15.7
G•C	70.5 ± 6.7•70.5 ± 6.7	43.2 ± 4.5•43.2 ± 4.5	60.7 ± 9.6•60.7 ± 9.6	52.0 ± 5.1•52.0 ± 5.1
G•C•Zn	31.0 ± 2.3•31.0 ± 2.3•10.3 ± 0.8	23.6 ± 1.6•23.6 ± 1.6•7.9 ± 0.5	61.5 ± 4.9•61.5 ± 4.9•20.5 ± 1.6	30.5 ± 1.9•30.5 ± 1.9•10.2 ± 0.6
Cis, Ciaplatin	3.4 ± 0.8	3.5 ± 0.8	5.3 ± 0.8	5.3 ± 0.8
5FU, 5-Fluorouracil	6.4 ± 0.6	16.5 ± 1.2	9.8 ± 0.5	9.9 ± 0.8
Mem, Memantine	55.2 ± 3.4	67.9 ± 2.5	68.7 ± 2.5	69.3 ± 6.8
TRZ, Thioridazine	8.8 ± 0.9	8.6 ± 1.1	7.9 ± 0.9	13.5 ± 1.3

**Figure 4:**
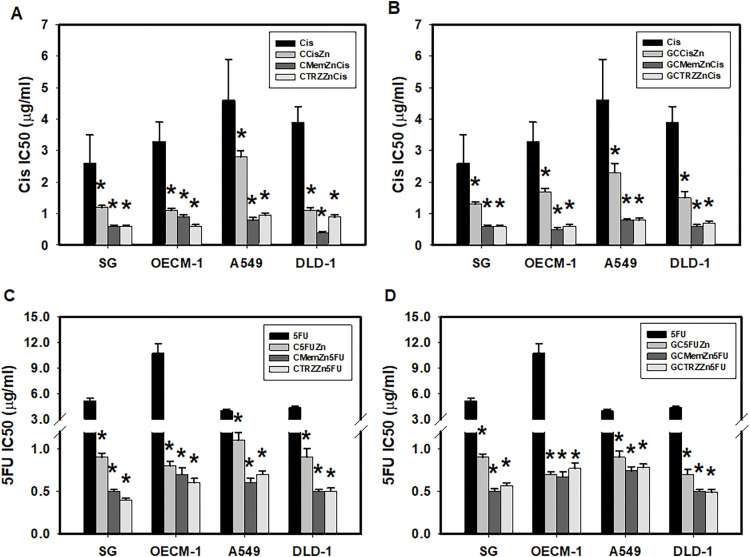
Potencies (IC_50_) of cisplatin (Cis, A, B) and 5-fluorouracil (5FU, C, D) on Na^+^-K^+^-Mg^2+^-ATPase inhibition in cultured cells. *p < 0.05.

**Figure 5:**
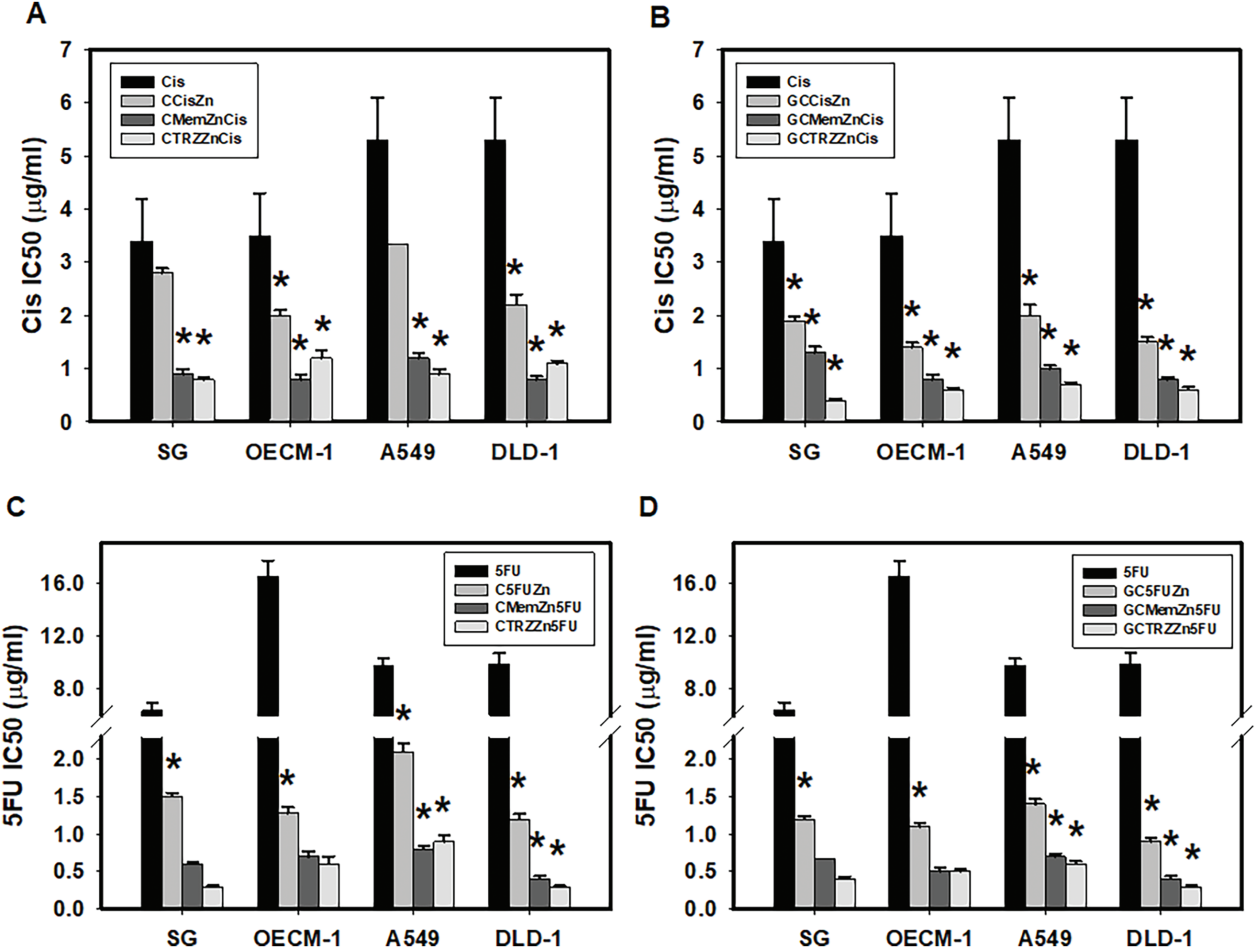
Potencies (IC_50_) of cisplatin (Cis, A, B) and 5-fluorouracil (5FU, C, D) on Mg^2+^-ATPase inhibition in cultured cells. *p < 0.05.

Notably, the novel combination regimens of C(GC)•Mem(TRZ)•Zn exhibited a synergistic enhancement (CI < 1) of selective anti-ATPase efficacy (EI > 1) for Cis and 5FU against both Na^+^-K^+^-Mg^2+^ATPase (Table 4, Fig. 4) and Mg^2+^-ATPase (Table 5, Fig. 5) in the aforementioned cancer cell lines. Among these combinations, the most effective regimen of GC•Mem(TRZ)•Zn significantly increased the inhibitory efficacy of Cis on Na^+^-K^+^-Mg^2+^-ATPase (Table 4A) and 5FU (Table 4B) by 4–7 and 9–15 fold, respectively. Furthermore, the enhancements observed for Mg^2+^-ATPase inhibition by Cis (Table 5A) and 5FU (Table 5B) were greater than 3–9 and 10–33 fold, respectively. The superior selective inhibitory effects of GC•Mem (TRZ)•Zn agents to those of C•Mem (TRZ)•Zn (Fig. 4A,C and Fig. 5A,C) are clearly illustrated (Fig. 4B,D, and Fig. 5B,D).

**Table 4:** PTM regimens enhance potency (IC_50_), synergism (CI < 1), and efficacy (EI) of Na^+^-K^+^-Mg^2+^-ATPase inhibition induced by cisplatin (A) and 5-fluorouracil (B) on cultured cells.

(A)						
Drugs	SG			OECM-1		
	IC_50_ (μg/mL; Zn^2+^, μM)	CI	EI^†^	IC_50_ (μg/mL; Zn^2+^, μM)	CI	EI^†^
C•Cis•Zn	35.4 ± 1.9•1.2 ± 0.06•11.8 ± 0.6	1.5	2*	33.7 ± 2.2•1.1 ± 0.07•11.2 ± 0.7	0.9	3*
C•Mem•Zn•Cis	18.6 ± 1.1•18.6 ± 1.1•6.2 ± 0.4•0.6 ± 0.04	1.1	4*	26.2 ± 2.0•26.2 ± 2.0•8.7 ± 0.7•0.9 ± 0.07	1.0	4*
C•TRZ•Zn•Cis	19.3 ± 1.0•1.9 ± 0.1•6.4 ± 0.3•0.6 ± 0.03	1.1	4*	19.3 ± 1.4•1.9 ± 0.14•6.4 ± 0.5•0.6 ± 0.05	0.8	6*
G•C•Cis•Zn	38.0 ± 2.0•38.0 ± 2.0•1.3 ± 0.07•12.7 ± 0.7	1.2	2*	51.2 ± 3.6•51.2 ± 3.6•1.7 ± 0.1•17.1 ± 1.2	1.5	2*
G•C•Mem•Zn•Cis	19.2 ± 1.2•19.2 ± 1.2•19.2 ± 1.2•6.4 ± 0.4•0.6 ± 0.04	0.9	4*	14.4 ± 2.1•14.4 ± 2.1•14.4 ± 2.1•4.8 ± 0.7•0.5 ± 0.07	0.6	7*
G•C•TRZ•Zn•Cis	18.3 ± 1.1•18.3 ± 1.1•1.8 ± 0.11•6.1 ± 0.4•0.6 ± 0.04	0.8	4*	18.6 ± 1.9•18.6 ± 1.9•1.9 ± 0.19•6.2 ± 0.6•0.6 ± 0.06	0.8	6*

Drugs	A549			DLD-1		
	IC_50_ (μg/mL; Zn^2+^, μM)	CI	EI^†^	IC_50_ (μg/mL; Zn^2+^, μM)	CI	EI^†^
C•Cis•Zn	83.8 ± 7.2•2.8 ± 0.2•27.9 ± 2.4	0.9	2*	33.7 ± 3.3•1.1 ± 0.1•11.2 ± 1.1	0.5	4*
C•Mem•Zn•Cis	23.2 ± 2.5•23.2 ± 2.5•7.7 ± 0.8•0.8 ± 0.08	0.5	6*	11.8 ± 1.1•11.8 ± 1.1•3.9 ± 0.4•0.4 ± 0.04	0.4	10*
C•TRZ•Zn•Cis	28.6 ± 2.0•2.9 ± 0.2•9.5 ± 0.7•0.95 ± 0.07	0.6	5*	27.3 ± 1.7•2.7 ± 0.17•9.1 ± 0.6•0.9 ± 0.06	0.7	4*
G•C•Cis•Zn	68.4 ± 7.6•68.4 ± 7.6•2.3 ± 0.3•22.8 ± 2.5	1.5	2*	45.7 ± 4.6•45.7 ± 4.6•1.5 ± 0.2•15.2 ± 1.5	1.1	3*
G•C•Mem•Zn•Cis	25.0 ± 0.8•25.0 ± 0.8•25.0 ± 0.8•8.3 ± 0.3•0.8 ± 0.03	0.8	6*	18.6 ± 1.6•18.6 ± 1.6•18.6 ± 1.6•6.2 ± 0.5•0.6 ± 0.05	0.8	7*
G•C•TRZ•Zn•Cis	24.5 ± 1.7•24.5 ± 1.7•2.4 ± 0.17•8.2 ± 0.6•0.8 ± 0.06	0.8	6*	22.0 ± 2.1•22.0 ± 2.1•2.2 ± 0.21•7.3 ± 0.7•0.7 ± 0.07	0.8	6*
^†^EI of Cis in each regimen is indicated. *The increased EI values are statistically significants *p* < 0.05.						

(B)						
Drugs	SG			OECM-1		
	IC_50_ (μg/mL; Zn^2+^, μM)	CI	EI^†^	IC_50_ (μg/mL; Zn^2+^, μM)	CI	EI^†^
C•5FU•Zn	28.1 ± 1.4•0.9 ± 0.05•9.4 ± 0.5	1.0	6*	23.6 ± 1.4•0.8 ± 0.05•7.9 ± 0.5	0.5	14*
C•Mem•Zn•5FU	14.2 ± 0.6•14.2 ± 0.6•4.7 ± 0.2•0.5 ± 0.02	0.8	10*	20.4 ± 2.5•20.4 ± 2.5•6.8 ± 0.8•0.7 ± 0.08	0.6	15*
C•TRZ•Zn•5FU	11.1 ± 0.7•1.1 ± 0.07•3.7 ± 0.2•0.4 ± 0.02	0.6	13*	16.7 ± 1.5•1.7 ± 0.15•5.6 ± 0.5•0.6 ± 0.05	0.6	18*
G•C•5FU•Zn	26.8 ± 1.2•26.8 ± 1.2•0.9 ± 0.04•8.9 ± 0.4	0.6	6*	22.2 ± 0.9•22.2 ± 0.9•0.7 ± 0.03•7.4 ± 0.3	0.5	15*
G•C•Mem•Zn•5FU	15.0 ± 1.0•15.0 ± 1.0•15.0 ± 1.0•5.0 ± 0.3•0.5 ± 0.03	0.6	10*	20.2 ± 1.9•20.2 ± 1.9•20.2 ± 1.9•6.7 ± 0.6•0.7 ± 0.06	0.7	15*
G•C•TRZ•Zn•5FU	17.0 ± 1.0•17.0 ± 1.0•1.7 ± 0.1•5.7 ± 0.3•0.6 ± 0.03	0.6	9*	23.1 ± 1.7•23.1 ± 1.7•2.3 ± 0.17•7.7 ± 0.6•0.8 ± 0.06	0.8	14*

Drugs	A549			DLD-1		
	IC_50_ (μg/mL; Zn^2+^, μM)	CI	EI^†^	IC_50_ (μg/mL; Zn^2+^, μM)	CI	EI^†^
C•5FU•Zn	33.6 ± 2.6•1.1 ± 0.09•11.2 ± 0.9	0.4	4*	26.1 ± 3.0•0.9 ± 0.1•8.7 ± 1.0	0.4	5*
C•Mem•Zn•5FU	17.8 ± 1.5•17.8 ± 1.5•5.9 ± 0.5•0.6 ± 0.05	0.4	7*	14.7 ± 0.7•14.7 ± 0.7•4.9 ± 0.2•0.5 ± 0.02	0.5	9*
C•TRZ•Zn•5FU	19.8 ± 1.3•2.0 ± 0.13•6.6 ± 0.4•0.7 ± 0.04	0.4	6*	15.9 ± 1.1•1.6 ± 0.11•5.3 ± 0.4•0.5 ± 0.04	0.4	9*
G•C•5FU•Zn	27.0 ± 2.1•27.0 ± 2.1•0.9 ± 0.07•9.0 ± 0.7	0.6	4*	20.0 ± 1.9•20.0 ± 1.9•0.7 ± 0.06•6.7 ± 0.6	0.5	6*
G•C•Mem•Zn•5FU	22.1 ± 1.4•22.1 ± 1.4•22.1 ± 1.4•7.4 ± 0.5•0.7 ± 0.05	0.8	6*	16.1 ± 0.7•16.1 ± 0.7•16.1 ± 0.7•5.4 ± 0.2•0.5 ± 0.02	0.7	9*
G•C•TRZ•Zn•5FU	23.4 ± 1.3•23.4 ± 1.3•2.3 ± 0.13•7.8 ± 0.4•0.8 ± 0.04	0.8	5*	14.8 ± 1.0•14.8 ± 1.0•1.5 ± 0.1•4.9 ± 0.3•0.5 ± 0.03	0.5	9*
^†^EI of 5FU in each regimen is indicated. *The increased EI values are statistically significants *p* < 0.05.						

**Table 5:** PTM regimens enhance potency (IC_50_), synergism (CI < 1), and efficacy (EI) of Mg^2+^-ATPase inhibition induced by cisplatin (A) and 5-fluorouracil (B) on cultured cells.

(A)						
Drugs	SG			OECM-1		
	IC_50_ (μg/mL; Zn^2+^, μM)	CI	EI^†^	IC_50_ (μg/mL; Zn^2+^, μM)	CI	EI^†^
C•Cis•Zn	84.5 ± 2.6•2.8 ± 0.09•28.1 ± 0.9	2.2	1	59.8 ± 3.2•2.0 ± 0.1•19.9 ± 1.1	1.6	2*
C•Mem•Zn•Cis	27.8 ± 2.8•27.8 ± 2.8•9.3 ± 0.9•0.9 ± 0.09	1.2	4*	24.5 ± 3.0•24.5 ± 3.0•8.2 ± 1.0•0.8 ± 0.1	1.0	4*
C•TRZ•Zn•Cis	24.0 ± 1.2•2.4 ± 0.12•8.0 ± 0.4•0.8 ± 0.04	0.9	4*	35.7 ± 4.1•3.6 ± 0.41•11.9 ± 1.4•1.2 ± 0.14	1.4	3*
G•C•Cis•Zn	57.2 ± 2.8•57.2 ± 2.8•1.9 ± 0.09•19.1 ± 0.9	1.4	2*	42.0 ± 2.7•42.0 ± 2.7•1.4 ± 0.09•14.0 ± 0.9	1.4	3*
G•C•Mem•Zn•Cis	39.0 ± 3.4•39.0 ± 3.4•39.0 ± 3.4•13.0 ± 1.1•1.3 ± 0.11	1.7	3*	23.8 ± 2.7•23.8 ± 2.7•23.8 ± 2.7•7.9 ± 0.9•0.8 ± 0.09	1.2	4*
G•C•TRZ•Zn•Cis	13.2 ± 0.9•13.2 ± 0.9•1.3 ± 0.09•4.4 ± 0.3•0.4 ± 0.03	0.5	9*	16.8 ± 1.2•16.8 ± 1.2•1.7 ± 0.12•5.6 ± 0.4•0.6 ± 0.04	0.8	6*

Drugs	A549			DLD-1		
	IC_50_ (μg/mL; Zn^2+^, μM)	CI	EI^†^	IC_50_ (μg/mL; Zn^2+^, μM)	CI	EI^†^
C•Cis•Zn	>100•3.33•33.3	>1.3	<2	67.2 ± 6.4•2.2 ± 0.2•22.4 ± 2.1	0.7	2*
C•Mem•Zn•Cis	36.4 ± 3.1•36.4 ± 3.1•12.1 ± 1.0•1.2 ± 0.1	1.0	4*	23.3 ± 1.7•23.3 ± 1.7•7.8 ± 0.6•0.8 ± 0.06	0.6	7*
C•TRZ•Zn•Cis	26.3 ± 2.8•2.6 ± 0.28•8.8 ± 0.9•0.9 ± 0.09	0.7	6*	31.8 ± 1.5•3.2 ± 0.15•10.6 ± 0.5•1.1 ± 0.05	0.6	5*
G•C•Cis•Zn	59.1 ± 4.6•59.1 ± 4.6•2.0 ± 0.2•19.7 ± 1.5	1.4	3*	45.4 ± 3.3•45.4 ± 3.3•1.5 ± 0.1•15.1 ± 1.1	1.2	4*
G•C•Mem•Zn•Cis	28.8 ± 1.9•28.8 ± 1.9•28.8 ± 1.9•9.6 ± 0.6•1.0 ± 0.06	1.1	5*	23.0 ± 1.1•23.0 ± 1.1•23.0 ± 1.1•7.7 ± 0.4•0.8 ± 0.04	1.0	7*
G•C•TRZ•Zn•Cis	21.8 ± 1.2•21.8 ± 1.2•2.2 ± 0.12•7.3 ± 0.4•0.7 ± 0.04	0.8	8*	17.6 ± 1.5•17.6 ± 1.5•1.8 ± 0.15•5.9 ± 0.5•0.6 ± 0.05	0.6	9*
^†^EI of Cis in each regimen is indicated. *The increased EI values are statistically significants *p* < 0.05.						

(B)						
Drugs	SG			OECM-1		
	IC_50_ (μg/mL; Zn^2+^, μM)	CI	EI^†^	IC_50_ (μg/mL; Zn^2+^, μM)	CI	EI^†^
C•5FU•Zn	46.0 ± 1.5•1.5 ± 0.05•15.3 ± 0.5	1.0	4*	38.4 ± 2.3•1.3 ± 0.08•12.8 ± 0.8	0.8	13*
C•Mem•Zn•5FU	17.0 ± 0.8•17.0 ± 0.8•5.7 ± 0.3•0.6 ± 0.03	0.7	11	22.1 ± 2.2•22.1 ± 2.2•7.4 ± 0.7•0.7 ± 0.07	0.8	24
C•TRZ•Zn•5FU	9.5 ± 0.3•0.95 ± 0.03•3.2 ± 0.1•0.3 ± 0.01	0.3	21	17.2 ± 2.9•1.7 ± 0.29•5.7 ± 1.0•0.6 ± 0.1	0.5	28
G•C•5FU•Zn	37.1 ± 1.1•37.1 ± 1.1•1.2 ± 0.04•12.4 ± 0.4	0.8	5*	33.1 ± 1.6•33.1 ± 1.6•1.1 ± 0.05•11.0 ± 0.5	0.9	15*
G•C•Mem•Zn•5FU	>20•20•20•6.7•0.67	0.8	10	16.0 ± 1.6•16.0 ± 1.6•16.0 ± 1.6•5.3 ± 0.5•0.5 ± 0.05	0.7	33
G•C•TRZ•Zn•5FU	10.1 ± 0.9•10.1 ± 0.9•1.0 ± 0.09•3.4 ± 0.3•0.4 ± 0.03	0.3	16	15.8 ± 1.1•15.8 ± 1.1•1.6 ± 0.11•5.3 ± 0.4•0.5 ± 0.04	0.6	33

Drugs	A549			DLD-1		
	IC_50_ (μg/mL; Zn^2+^, μM)	CI	EI^†^	IC_50_ (μg/mL; Zn^2+^, μM)	CI	EI^†^
C•5FU•Zn	62.1 ± 2.9•2.1 ± 0.1•20.7 ± 1.0	0.6	5*	35.8 ± 2.0•1.2 ± 0.07•11.9 ± 0.7	0.3	8*
C•Mem•Zn•5FU	23.5 ± 1.1•23.5 ± 1.1•7.8 ± 0.4•0.8 ± 0.04	0.6	12*	11.0 ± 1.1•11.0 ± 1.1•3.7 ± 0.4•0.4 ± 0.04	0.2	25*
C•TRZ•Zn•5FU	25.8 ± 2.4•2.6 ± 0.24•8.6 ± 0.8•0.9 ± 0.08	0.6	11*	10.1 ± 0.4•1.0 ± 0.04•3.4 ± 0.1•0.3 ± 0.01	0.1	33*
G•C•5FU•Zn	43.4 ± 2.2•43.4 ± 2.2•1.4 ± 0.07•14.5 ± 0.7	0.9	7*	26.6 ± 1.4•26.6 ± 1.4•0.9 ± 0.05•8.9 ± 0.5	0.6	11*
G•C•Mem•Zn•5FU	19.9 ± 1.3•19.9 ± 1.3•19.9 ± 1.3•6.6 ± 0.4•0.7 ± 0.04	0.7	14*	11.6 ± 1.1•11.6 ± 1.1•11.6 ± 1.1•3.9 ± 0.4•0.4 ± 0.04	0.4	25*
G•C•TRZ•Zn•5FU	16.7 ± 1.3•16.7 ± 1.3•1.7 ± 0.13•5.6 ± 0.4•0.6 ± 0.04	0.6	16*	8.6 ± 0.5•8.6 ± 0.5•0.9 ± 0.05•2.9 ± 0.2•0.3 ± 0.02	0.3	33*
^†^EI of 5FU in each regimen is indicated. *The increased EI values are statistically significants *p* < 0.05.						

### Antibacterial Effects

**Figure 6:**
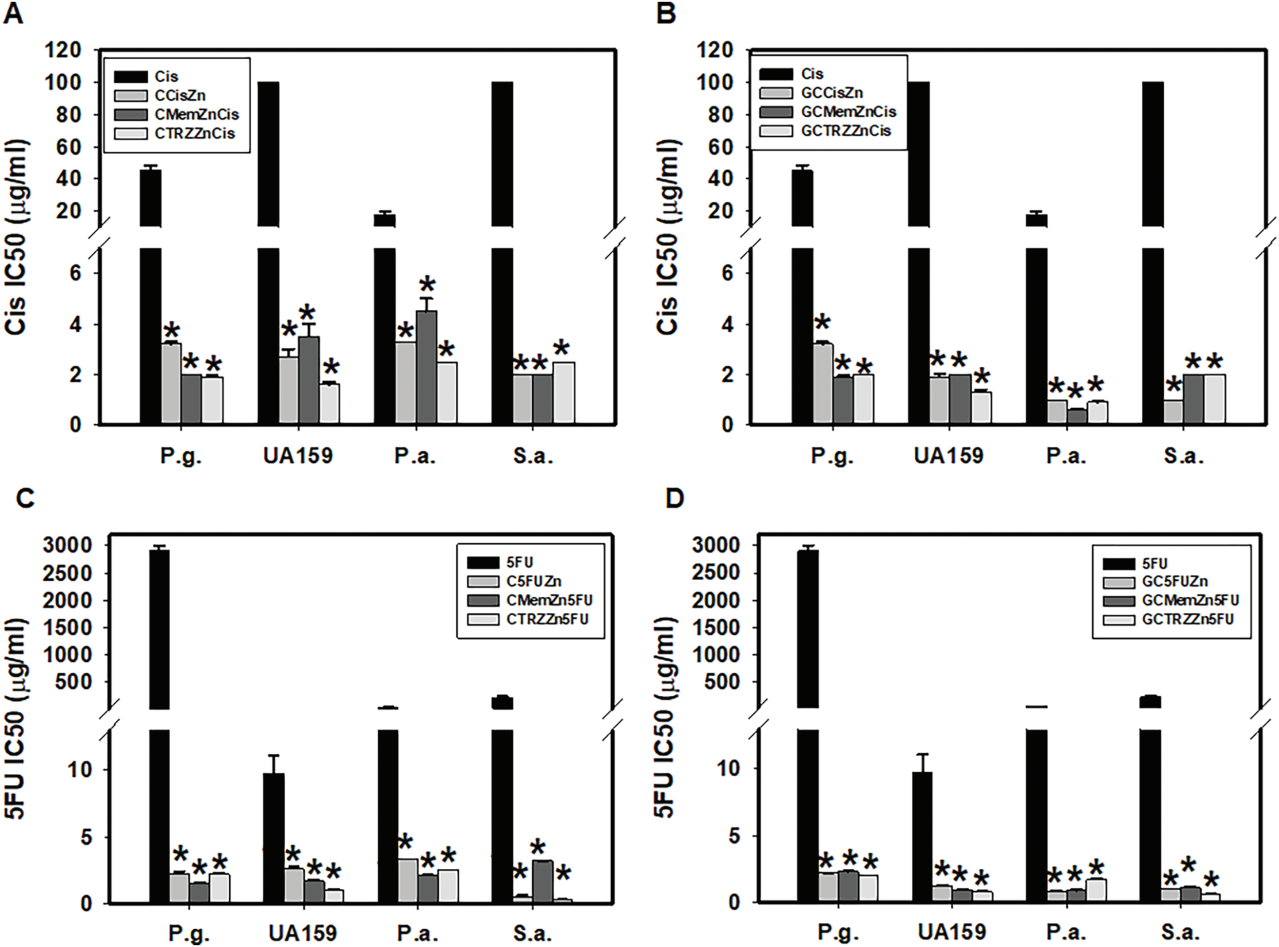
Antibacterial potency (IC_50_) and synergism (CI < 1) of cisplatin (Cis, A, B) and 5-fluorouracil (5FU, C, D) on four cultured pathogenic biofilms. P.g., *Porphyromonas gingivalis*, UA159, *Streptococcus mutans*, P.a., *Pseudomonas aeruginosa* and S.a., *Staphylococcus aureus*. *p < 0.05.

As indicated in Table 6A, the antibacterial effects of the drugs administered alone against four cultured pathogenic biofilms were predominantly weak, with the exception of Cis on *Pseudomonas aeruginosa* (P.a.), 5FU on *Streptococcus mutans* (UA159) and P.a., and TRZ on *Porphyromonas gingivalis* (P.g.), UA159, and *Staphylococcus aureus* (S.a.), respectively. In contrast, the combination regimens involving Mem or TRZ demonstrated predominantly synergistic antibacterial effects (CI < 1), with efficacy index (EI) increasing by factors of 2–3, 5–6,6–58 and 4–10 on P.g., UA159, P.a., and S.a., respectively (Table 6B). Notably, these combination regimens significantly augmented the antibacterial efficacy of Cis, yielding increases of 14–24, 29–77, 4–29, and 50–100 fold against P.g., UA159, P.a., and S.a., respectively (Table 7A, Fig. 6A,B). Similarly, the efficacy of 5FU was enhanced by factors ranging from 1264–1939, 4–12, 15–61, and 70–743 fold, respectively (Table 7B, Fig. 6C,D).

**Table 6:** Antibacterial potencies (IC_50_) of drugs administered individually on four cultured pathogenic biofilms.

(A) Drug alone				
Drugs	IC_50_ (μg/mL; Zn^2+^, μM)			
	P.g.	UA159	P.a.	S.a.
C,Curcumin	122.9 ± 7.9	251 ± 9.5	602.3 ± 11.3	238.3 ± 11.3
G, Green tea polyphenol	125 ± 12.7	446.5 ± 18.5	183.9 ± 27.8	>1000
Zn, ZnSO_4_	501.0 ± 41.6	1858.3 ± 72.5	>3000	2035.4 ± 229
Cis, Ciaplatin	44.9 ± 3.4	>100	17.4 ± 2.3	>100
5FU, 5-Fluorouracil	2907.8 ± 97.1	9.7 ± 1.4	49 ± 2	223.0 ± 20.8
Mem, Memantine	175.5 ± 11.2	478.5 ± 37.5	280.7 ± 22.8	457.7 ± 9
TRZ, Thioridazine	20 ± 0.5	24.5 ± 0.6	>300	35.6 ± 5.6

(B) PTM regimens						
Drugs	P.g.			UA159		
	IC_50_ (μg/mL; Zn^2+^, μM)	CI^†^	EI^‡^	IC_50_ (μg/mL; Zn^2+^, μM)	CI	EI
G•C	92.7 ± 7.0•92.7 ± 7.0	1.5		95.5 ± 4.5•95.5 ± 4.5	0.6	
G•C•Zn	85.7 ± 7.2•85.7 ± 7.2•26.3 ± 2.1	1.0		55.5 ± 1.5•55.5 ± 1.5•16.5 ± 0.5	0.6	
C•Mem•Zn	59.6 ± 4.9•59.6 ± 4.9•18.6 ± 1.4	0.9	3*	78 ± 10•78 ± 10•23 ± 3	0.5	6*
C•TRZ•Zn	82.6 ± 2.1•8.3 ± 0.2•26.3 ± 0.8	1.1	2*	50.1 ± 1.2•5 ± 0.1•15.8 ± 0.5	0.4	5*
G•C•Mem•Zn	67.6 ± 5.7•67.6 ± 5.7•67.6 ± 5.7•22.4 ± 1.8	1.2	3*	78.2 ± 6•78.2 ± 6•78.2 ± 6•26.1 ± 2.1	1	6*
G•C•TRZ•Zn	69 ± 2.6•69 ± 2.6•6.9 ± 0.3•23 ± 0.9	1.1	3*	40.1 ± 4.5•40.1 ± 4.5•4.1 ± 0.5•13.3 ± 1.4	0.6	6*
G•C	149 ± 9•149 ± 9	1.1		132.5 ± 3.5•132.5 ± 3.5	0.7	
G•C•Zn	>100•100•30	>0.7		70.5 ± 1.5•70.5 ± 1.5•21.5 ± 0.5	0.6	
C•Mem•Zn	43.7 ± 2.8•43.7 ± 2.8•13 ± 1.0	0.2	*	>100•100•33	>0.7	<5*
C•TRZ•Zn	91.1 ± 5.6•9.1 ± 0.6•27.3 ± 1.7	0.2	3*	84.2 ± 1.2•8.3 ± 0.3•26.6 ± 1.6	0.6	4*
G•C•Mem•Zn	20.4 ± 4.6•20.4 ± 4.6•20.4 ± 4.6•6.7 ± 1.4	0.2	4*	47.3 ± 4.3•47.3 ± 4.3•47.3 ± 4.3•15.8 ± 1.4	0.5	10*
G•C•TRZ•Zn	51.9 ± 5.1•51.9 ± 5.1•5.2 ± 0.5•17.3 ± 1.7	0.4	8*	57.9 ± 1•57.9 ± 1•5.9 ± 0•19.4 ± 0.2	0.6	6*
P.g., *Porphyromonas gingivalis*; UA159, *Streptococcus mutan*s; P.a., *Pseudomonas aeruginosa*; S.a., *Staphylococcus aureus*.^†^CI and ^‡^EI were calculated using the equations described in the Methods section. EI of Mem or TRZ in each regimen is indicated. *The increased EI values are statistically significants *p* < 0.05.						

**Table 7:** Antibacterial potencies (IC_50_), synergism (CI < 1), and increased efficacies (EI) of various PTM regimens on four cultured pathogenic biofilms

(A) Cisplatin						
Drugs	P.g.			UA159		
	IC_50_ (μg/mL; Zn^2+^, μM)	CI	EI^†^	IC_50_ (μg/mL; Zn^2+^, μM)	CI	EI
C•Cis•Zn	104.9 ± 1.2•3.2 ± 0.1•32.4 ± 0.8	1.0	14*	88.1 ± 6.7•2.7 ± 0.3•27.1 ± 2.6	0.4	37*
C•Mem•Zn•Cis	49 ± 1.0•49 ± 1.0•16.5 ± 0.5•2 ± 0	0.8	23*	97.5 ± 7.5•97.5 ± 7.5•32.5 ± 2.5•3.5 ± 0.5	0.6	29*
C•TRZ•Zn•Cis	55.6 ± 1.2•5.6 ± 0.1•18.5 ± 0.4•1.9 ± 0.04	0.8	24*	49.4 ± 1.6•4.9 ± 0.2•16.5 ± 0.5•1.6 ± 0.1	0.4	63*
G•C•Cis•Zn	94.4 ± 3.4•94.4 ± 3.4•3.2 ± 0.1•31.5 ± 1.1	1.2	14*	50 ± 2.9•50 ± 2.9•1.9 ± 0.1•16.8 ± 1	0.6	53*
G•C•Mem•Zn•Cis	57.3 ± 1.4•57.3 ± 1.4•57.3 ± 1.4•19.1 ± 0.5•1.9 ± 0.05	1.0	24*	>60•60•6•20•2	>0.7	<50*
G•C•TRZ•Zn•Cis	>60•60•6•20•2	>1.0	<23*	40.3 ± 1.6•40.3 ± 1.6•4 ± 0.2•13.4 ± 0.5•1.3 ± 0.1	0.6	77*

Drugs	P.a.			S.a.		
	IC_50_ (μg/mL; Zn^2+^, μM)	CI	EI	IC_50_ (μg/mL; Zn^2+^, μM)	CI	EI
C•Cis•Zn	>100•3.3•33	>0.4	<5*	62.1 ± 6.1•2.0 ± 0.0•18.7 ± 1.7	0.3	50*
C•Mem•Zn•Cis	126 ± 18•126 ± 18•42 ± 6•4.5 ± 0.5	0.9	4*	47 ± 1•47 ± 1•15.5 ± 0.5•2 ± 0	0.3	50*
C•TRZ•Zn•Cis	>75•7.5•25•2.5	>0.3	<7*	>75•7.5•25•2.5	>0.6	<40*
G•C•Cis•Zn	32.7 ± 3.4•32.7 ± 3.4•1.0 ± 0.0•10.9 ± 1.1	0.3	17*	33.0 ± 2.0•33.0 ± 2.0•1.0 ± 0.0•11.0 ± 1.0	0.3	100*
G•C•Mem•Zn•Cis	18.2 ± 0.8•18.2 ± 0.8•18.2 ± 0.8•6.1 ± 0.3•0.6 ± 0.03	0.2	29*	>60•60•6•20•2	>0.5	<50*
G•C•TRZ•Zn•Cis	27 ± 1.1•27 ± 1.1•2.7 ± 0.1•9 ± 0.4•0.9 ± 0.04	0.2	19*	>60•60•6•20•2	>0.7	<50*
^†^EI of Cis in each regimen is indicated. *The increased EI values are statistically significants *p* < 0.05.						

(B) 5-fluorouracil						
Drugs	P.g.			UA159		
	IC_50_ (μg/mL; Zn^2+^, μM)	CI	EI^†^	IC_50_ (μg/mL; Zn^2+^, μM)	CI	EI
C•5FU•Zn	66.6 ± 5.3•2.2 ± 0.2•22 ± 1.7	0.6	1322*	78.6 ± 5.4•2.6 ± 0.2•26 ± 1.8	0.6	4*
C•Mem•Zn•5FU	44.2 ± 1.8•44.2 ± 1.8•14.7 ± 0.6•1.5 ± 0.1	0.6	1939*	51.4 ± 2.6•51.4 ± 2.6•17.1 ± 0.9•1.7 ± 0.1	0.5	6*
C•TRZ•Zn•5FU	66.3 ± 2.5•6.6 ± 0.3•22.1 ± 0.8•2.2 ± 0.1	0.9	1322*	31 ± 3.2•3.1 ± 0.3•10.3 ± 1.1•1.0 ± 0.1	0.4	10*
G•C•5FU•Zn	64.5 ± 1.0•64.5 ± 1.0•2.2 ± 0.03•21.5 ± 0.3	0.7	1322*	35 ± 2.5•35 ± 2.5•1.2 ± 0.1•11.7 ± 0.8	0.5	8*
G•C•Mem•Zn•5FU	70.3 ± 4.4•70.3 ± 4.4•70.3 ± 4.4•23.4 ± 1.5•2.3 ± 0.1	1.2	1264*	25.6 ± 2.0•25.6 ± 2.0•25.6 ± 2.0•8.5 ± 0.7•0.9 ± 0.1	0.4	11*
G•C•TRZ•Zn•5FU	>60•60•6•20•2	>1.0	<1454*	24.2 ± 0.8•24.2 ± 0.8•2.4 ± 0.1•8.1 ± 0.3•0.8 ± 0.03	0.4	12*

Drugs	P.a.			S.a.		
	IC_50_ (μg/mL; Zn^2+^, μM)	CI	EI	IC_50_ (μg/mL; Zn^2+^, μM)	CI	EI
C•5FU•Zn	>100•3.3•33	>0.2	<15*	14.9 ± 2.8•0.5 ± 0.1•4.9 ± 0.9	0.1	446*
C•Mem•Zn•5FU	62.4 ± 1.1•62.4 ± 1.1•20.8 ± 0.4•2.1 ± 0.04	0.4	23*	96.4 ± 0.3•96.4 ± 0.3•32.1 ± 0.1•3.2 ± 0.01	0.6	70*
C•TRZ•Zn•5FU	>75•7.5•25•2.5	>0.2	<20*	7.7 ± 0.1•0.8 ± 0.01•2.6 ± 0.04•0.3 ± 0.004	0.1	743*
G•C•5FU•Zn	23.3 ± 1.9•23.3 ± 1.9•0.8 ± 0.1•7.8 ± 0.6	0.2	61*	32.6 ± 1.5•32.6 ± 1.5•1 ± 0•10.7 ± 0.3	0.3	223*
G•C•Mem•Zn•5FU	27.6 ± 1.8•27.6 ± 1.8•27.6 ± 1.8•9.2 ± 0.6•0.9 ± 0.1	0.3	54*	31.7 ± 3.6•31.7 ± 3.6•31.7 ± 3.6•10.6 ± 1.2•1.1 ± 0.1	0.3	203*
G•C•TRZ•Zn•5FU	49.6 ± 1.5•49.6 ± 1.5•5.0 ± 0.2•16.5 ± 0.5•1.7 ± 0.1	0.4	29*	17.7 ± 1.5•17.7 ± 1.5•1.8 ± 0.1•5.9 ± 0.5•0.6 ± 0.05	0.2	372*
^†^EI of 5FU in each regimen is indicated. *The increased EI values are statistically significants *p* < 0.05.						

## Discussion

The increasing global incidence of cancer poses substantial challenges that require immediate attention and innovative solutions [2]. Carcinogenesis is influenced by a multitude of risk factors, including oxidative stress, inflammation, immunodeficiency, and gastrointestinal dysbiosis. Recognizing the pivotal role of chemoprevention in cancer management, our research team has dedicated over thirty years to exploring the chemopreventive properties of phytopolyphenols [3,4]. Notably, novel combinations of curcumin (C) and green tea polyphenols (G), such as GC, have exhibited synergistic (CI < 1) and selective anticancer and antibacterial effects (Tables 1B and 6B). Clinical trials involving C and G, along with other tea polyphenols, have indicated their potential utility in cancer prevention [28,29].

Among various cancer types, non-small cell lung cancer and colon cancer are among the most prevalent worldwide, while oral cancers present significant health challenges in regions such as East Asia, India, and Taiwan [13,15,19]. In the current study, both cisplatin (Cis) and 5-fluorouracil (5FU), which are widely used chemotherapeutic agents, demonstrated non-selective inhibition of growth in oral (OECM-1), lung (A549), and colon (DLD-1) cancer cell lines. However, the severe side effects associated with these agents, including nephrotoxicity, neurotoxicity, and oral mucositis—affecting 60%–80% of patients—substantially impair quality of life and contribute to the discontinuation of cancer therapies [16,17]. Therefore, the development of novel regimens aimed at enhancing the selective anticancer efficacy of Cis and 5FU would be highly beneficial for patients suffering from these debilitating side effects.

In this study, we have demonstrated the synergistic anticancer effects of PTM regimens on the three cancer cell lines: oral cancer (OECM-1), non-small cell lung cancer (A549), and colon cancer (DLD-1). Additionally, preliminary experiments were conducted on SHSY5Y neuroblastoma cells, GL261 glioma cells, MCF7 breast cancer cells, and ARPE19 ocular epithelial cells, with the former two cancer cell lines exhibiting particular sensitivity to the anticancer effects of PTM regimens. We anticipate conducting extensive experiments on additional cancer cell lines. Furthermore, the four selected pathogens were investigated in these experiments due to their significant role in the pathogenesis of infectious diseases, including periodontitis (P.g.), dental caries (UA159), pneumonia (P.a.), and wound infections (S.a.), particularly in the context of hospital-acquired infections. We collaborated with Drs. Hong-Yunn Don and Tsai-Lin Yang from the National Institute of Infectious Diseases and Vaccinology, National Health Research Institute, Taiwan, to evaluate the antibacterial effects of PTM regimens. Preliminary results indicated that certain PTM regimens effectively inhibited the proliferation of 43300 *S. aureus* (MRSA), 51299 *E. faecalis* (VRE), and multidrug-resistant strains of *Mycobacterium tuberculosis* (TB) (KVGH 376 and KVGH 264). We sincerely hope that PTM regimens can be further explored in relation to a broader range of cancer cells and pathogens.

In this study, the application of PTM regimens was observed to synergistically enhance (CI < 1) the selective anticancer efficacy of Cis and 5FU, thereby suggesting a potentially effective therapeutic strategy. Specifically, the optimal regimens of GC•TRZ•Zn increased the selective anticancer efficacy of Cis by 7, 9, and 17 fold for OECM-1, A549, and DLD-1 cell lines, respectively. In contrast, the GC•Mem•Zn regimen enhanced the anticancer efficacy of 5FU by 5, 6, and 12 fold for the three cancer cell lines, respectively. On the other hand, both GC•Mem•Zn and GC•TRZ•Zn were superior to those NSAIDs-PTM regimens in terms of increasing anticancer selectivity and efficacy of Cis and 5FU in our previous report [25]. These results highlight the necessity for further clinical exploration. Given that all components of the PTM regimens are both clinically effective and safe, they have been recognized as innovative and creative solutions, resulting in the issuance of five patents for their application in the prevention and management of cancers, infectious diseases, dementia, chronic pain, and other conditions [6–8].

The challenge of multidrug resistance (MDR) in cancer chemotherapy remains a significant concern [30,31]. Recent research has indicated that the overexpression of ATPases in cancer cells plays a critical role in the development and progression of MDR [32,33]. Cardiac glycosides, such as digoxin, which inhibit Na^+^-K^+^-Mg^2+^-ATPase, have demonstrated efficacy as anticancer agents [34,35]. Furthermore, Na^+^-K^+^-Mg^2+^-ATPase located on cell membranes is recognized as an efflux pump for anticancer agents; thus, inhibitors of this enzyme have been found to effectively reverse MDR and enhance the anticancer effects of chemotherapeutic agents [36,37]. Additionally, inhibitors of mitochondrial Mg^2+^-ATPase, such as Ru-complexes [38,39], have been validated as potent anticancer agents. In our study, PTM regimens, whether administered alone or in conjunction with Cis and 5FU, demonstrated a synergistic inhibition of both Na^+^-K^+^-Mg^2+^-ATPase and Mg^2+^-ATPase activities, suggesting their potential mechanisms of action against cancer and their capacity to address the challenges posed by MDR [40].

Infectious diseases among cancer patients significantly contribute to morbidity and mortality. Therefore, enhancing the antibacterial properties of anticancer agents is crucial for improving cancer therapy outcomes. Our findings indicate that PTM regimens, particularly in combination with Cis and 5FU, exhibited notable antibacterial effects against four cultured pathogenic biofilms, warranting further clinical investigation.

While this study suggests that PTM regimens may serve as promising anticancer and antibacterial treatments, the experimental results are limited to *in vitro* cultured cancer cells and pathogens. It is imperative to further explore the mechanisms of action (including apoptosis, cell cycle analysis, clonogenic assays, metabolic experiments, and western blot analysis of target genes) and to conduct clinical trials for *in vivo* validation of PTM regimens in the near future.

## Conclusions

**Figure 7:**
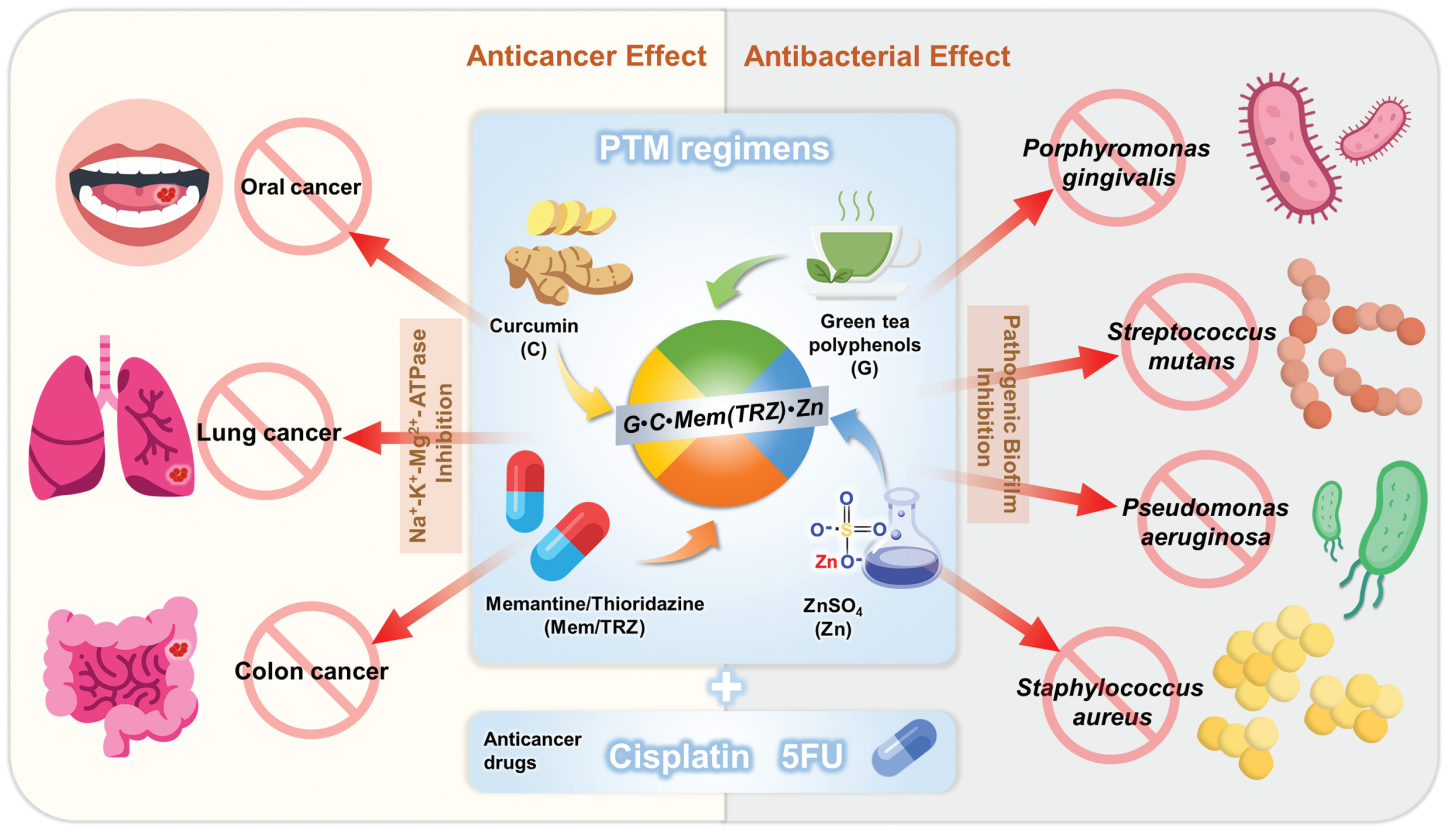
PTM regimens have potential antagonistic effects against the problem of multi-drug resistance in cancers and infectious diseases. G•C•Mem(TRZ)•Zn encompass three major categories of drugs: phytopolyphenols (green tea polyphenols and curcumin), repurposed drugs (memantine or thioridazine) and metal ions (ZnSO_4_). The novel PTM regimen can be combined with anticancer agents such as cisplatin and 5-fluorouracil (5FU), with the aim of achieving synergistic effects on three cancer cell lines (oral cancer OECM-1, lung cancer A549 and colorectal cancer DLD-1) as well as four pathogenic biofilms (*Porphyromonas gingivalis, Streptococcus mutans, Pseudomonas aeruginosa and Staphylococcus aureus*). Certain icons are reproduced from Flaticon.com with permission.

In conclusion, as depicted in Fig. 7, we have identified a promising therapeutic strategy involving novel PTM regimens composed of phytopolyphenols (P, curcumin and green tea polyphenols), targeting drugs (T, memantine, thioridazine, cisplatin, and 5-fluorouracil), and metals (M, ZnSO_4_). These regimens demonstrated remarkable synergistic effects (CI < 1) and selective anticancer efficacy across three cultured cancer cell lines, significantly enhancing the therapeutic potential of cisplatin and 5-fluorouracil. Importantly, PTM regimens synergistically inhibited Na^+^-K^+^-Mg^2+^-ATPase and Mg^2+^-ATPase in cancer cells, addressing key challenges associated with multidrug resistance in cancer therapy and infectious disease management. Furthermore, the synergistic antibacterial effects of PTM regimens against four cultured pathogenic biofilms underscore their potential to reduce the morbidity and mortality associated with infectious diseases in cancer patients. Given the clinical effectiveness and safety profiles of all drugs included in these regimens, further preclinical and clinical investigations are strongly encouraged to validate their efficacy and explore their broader applications in oncology and infectious disease therapeutics.

## Data Availability

The dataset used and analyzed during the current study are available from the corresponding author on reasonable request.
